# Apiaceae Family an Important Source of Petroselinic Fatty Acid: Abundance, Biosynthesis, Chemistry, and Biological Proprieties

**DOI:** 10.3390/biom13111675

**Published:** 2023-11-20

**Authors:** Ahmed Hajib, Said El Harkaoui, Hasnae Choukri, Aya Khouchlaa, Sarra Aourabi, Naoual El Menyiy, Abdelhakim Bouyahya, Bertrand Matthaeus

**Affiliations:** 1 Laboratory of Bioactive and Molecules of Interest, National Agency of Medicinal and Aromatic Plants (NAMAP), Taounate 34000, Morocco; 2 Department of Safety and Quality of Cereals, Max Rubner-Institut, Federal Research Insitute for Nutrition and Food, Schützenberg 12, D-32756 Detmold, Germany; 3 International Center for Agricultural Research in the Dry Areas, Rabat 10000, Morocco; 4 Laboratory of Biochemistry, National Agency of Medicinal and Aromatic Plants (NAMAP), Taounate 34000, Morocco; 5 Laboratory of Pharmacology, National Agency of Medicinal and Aromatic Plants (NAMAP), Taounate 34000, Morocco; 6 Laboratory of Human Pathologies Biology, Faculty of Sciences, Mohammed V University in Rabat, Rabat 10106, Morocco

**Keywords:** petroselinic acid, Apiaceae, lipid, biosynthesis, reactivity, biological activity

## Abstract

Petroselinic fatty acid (PeFA) is considered a rare fatty acid and one of the most important fatty acids in the Apiaceae family. Its content varies depending on plant species, geographical origin, extraction method, ripeness, etc. Indeed, reported levels of petroselinic fatty acid range from 10.4 to 75.6% (in anise seed oil), 1 to 81.9% (in coriander seed oil), 28.5 to 57.6% (in caraway seed oil), 49.4 to 75.6% (in celery seed oil), 41.3 to 61.8% (in caraway seed oil), 79.9 to 87.2% (in dill seed oil), 43.1 to 81.9% (in fennel seed oil), and 35 to 75.1% (parsley seed oil). In this review, we also show current knowledge about genes encoding biosynthesis, from the desaturation of 16:0-ACP to petroselinic acid stored in triacylglycerol in the seeds. Furthermore, petroselinic acid is not related to the synthesis of ABA. PeFA was successfully isolated from Apiaceae family plant seeds in order to study their reactivity and biological activities. Several investigations showed that this fatty acid has a wide range of biological potentials, including antidiabetic, antibacterial, and antifungal activities. In cosmetics, PeFA alone or in association with other active compounds has interesting applications as an anti-inflammatory agent for the treatment of skin, hair, and nail disorders.

## 1. Introduction

Infrequent natural fatty acids are mostly found in oil-rich seeds as storage compounds [1]. They typically exhibit a plant family-specific distribution [2]. For example, uncommon hydroxy fatty acids are found in the seed oil of some Euphorbiaceae [3,4]; acetylenic fatty acids are common in members of the family Santalaceae, Olacaceae, Asteraceae, and Caesalpiniaceae, respectively [5,6,7]; infrequent epoxy acids are identified in the Asteraceae and Cruciferae families [8,9,10]; cyclic fatty acids have been found in the Malvaceae family [11,12]; uncommon conjugated fatty acids have been reported in the seed oil of some Punicaceae, Euphorbiaceae, Bignoniaceae, and Asteraceae species [13,14]. Fatty acids with unusual unsaturation are widely distributed in the Apiaceae family [15,16].

Apiaceae species yield a wide variety of distinctive specialized metabolites (i.e., volatile phenylpropanoids, furanocoumarins, sesquiterpene coumarins, polyacetylenes, phthalides, etc.), some of them have been described as uncommon natural phytochemicals exclusive of this family, which offers a great potential for bioprospection [17,18,19,20]. Petroselinic fatty acid (C18:1 cis-Δ6) (PeFA) is a rare fatty acid, characteristic of the lipid fraction of Apiaceae family plants [16]. This fatty acid constitutes about 80% of the total fatty acids in the seeds of Apiaceae, while it is practically absent in the leaves and the other parts of these plants [21].

**Figure 1:**
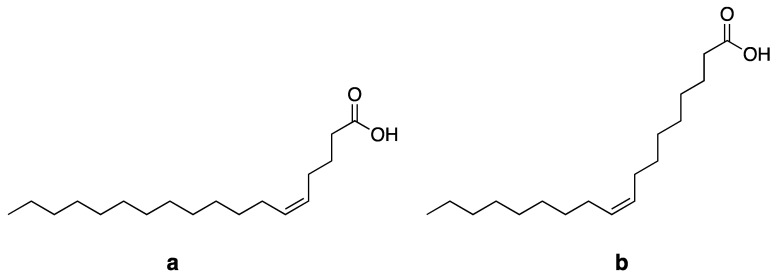
Structure of petroselinic acid (C18:1 cis-Δ6) (**a**), and oleic acid (C18:1 cis-Δ9) (**b**).

Petroselinic acid (18:1 cis-Δ6) or cis-6-octadecenoic acid (C_18_H_34_O_2_) is a positional isomer of oleic acid (18:1 cis-Δ9) (Figure 1) [16]. They differ in the position of the double bond; PeFA has a double bond at the position C_6_-C_7_, whereas oleic acid has a double bond at the position C_9_-C_10_ [22]. The location of the double bond has a significant influence on the physicochemical characteristics. For example, petroselinic acid has a melting temperature of 30 °C, whereas oleic acid melts already at 14 °C [23].

Petroselinic acid was detected for the first time in parsley (*Petroselinum crispum*) seed oil by Vongerichten and Kohler, then Scherer established the presence of this infrequent fatty acid in the seed oils of two other plants of the Apiaceae family (*Pimpinella anisum* and *Foeniculum capillaceum*) [24]. Afterward, many studies reported the presence of petroselinic acid in many plants of the Apiaceae family [16,25,26,27,28]. According to [29], PeFA was detected in some Araliaceae family seeds. It is also a minor constituent of the lipid fraction of basil (*Ocimum basilicum*) seeds and other plants [24,30,31,32]. Petroselinic acid content varies greatly from plant to plant; it can be influenced by climatic and soil conditions [33,34]. Many studies have also indicated that the content of PeFA also varies according to the maturity stage of the seeds [35,36].

In particular, petroselinic acid can be cleaved through oxidation of the double bond into lauric (12:0) and adipic (6:0) acids, both of which have important applications in the production of nylon and emollients, emulsifiers, detergents, and soap [37,38]. Adipic acid is also used in the food industry as an acidifier (E355) and as a substitute for tartaric acid in baking powder and lemonade [38]. Petroselinic acid has been the subject of several patents and scientific studies. It is used in the form of a tablet as an active compound for the treatment of inflammation of human superficial tissues and for the modulation of lipid metabolism. Petroselinic acid is also used in cosmetic formulations as a slimming and moisturizing agent, as well as an agent to reduce skin irritation in α-hydroxy acid-containing compositions. When combined with zinc (II) salts or with taurine, petroselinic acid is used for the prevention and control of hair ageing [22,39]. In addition, petroselinic acid holds a wide range of biological effects, such as antidiabetic, antibacterial, and antifungal properties [40,41,42]. Some studies have also investigated the biological properties of PeFA derivatives.

To the best of the authors’ knowledge, the last review about petroselinic fatty acid was conducted in 1962. Thus, in this context, this paper aimed to carry out a systematic literature review of petroselinic fatty acid scientific papers and patents during the last 66 years (from 1956 to 2022). Different research websites were used, including Scopus, Web of Science, Google Scholar, and Google Patent. This review investigated the natural sources, biosynthesis, identification and quantification, isolation, reactivity, and biological properties of petroselinic fatty acid.

## 2. Natural Sources of Petroselinic Acid

Species belonging to the Apiaceae family (also known as Umbelliferae) are an excellent natural source of petroselinic fatty acid, which is considered a rare fatty acid [1]. Apiaceae family consists of 3700 species, and many of them are used in different domains, including nutrition, cosmetics, and medicines. However, anise (*Pimpinella anisum*), caraway (*Carum carvi*), celery (*Apium graveolens*), coriander (*Coriandrum sativum*), cumin (*Cuminum cyminum*), dill (*Anethum graveolens*), fennel (*Foeniculum vulgar*), and parsley (*Petroselinum crispum*) are the most well-known, cultivated and economically significant members of the family [22]. Thus, in this review, we focused on these eight species as potential sources of petroselinic acid.

Different extraction methods were used for oil extraction from the species in which PeFA was the primary fatty acid (Table 1). Solvent extraction techniques result in higher oil yield but use hazardous solvents; supercritical fluid extraction uses nontoxic CO_2_ but requires higher energy [43]. In addition, the exchange of hazardous solvents with green ones, such as 2-methyltetrahydrofuran (MeTHF), has begun (Table 1).

The PeFA content changes according to plant species, geographical origins, extraction method, maturity stage, etc. Values recorded ranged between 10.4 and 75.6% (anise), 1 and 81.9% (coriander), 28.5 and 57.6% (caraway), 49.4 and 75.6% (celery), 41.3 and 61.8% (cumin), 79.9 and 87.2% (dill), 43.1 and 81.9% (fennel), and 35 and 75.1% (parsley) (Table 1). Ngo-Duy, Destaillats, Keskitalo, Arul, and Angers [56] analyzed caraway, celery, and parsley oils for their triacylglycerol (TAG) composition and fatty acid distribution between the sn-1(3) and sn-2 positions of TAG. The authors identified 22 TAGs in the tested samples with a predominance of glyceryl tripetroselinate in celery and parsley seed oils, with levels ranging from 38.7 to 55.3%. However, caraway seed oil contained 11.4% only [56]. The regiospecificity showed a non-random distribution of different fatty acids, with sn-1(3) as a predominant location of PeFA in celery and parsley oils. In contrast, PeFA was mainly located at the sn-2 position in caraway seed oil [56].

Petroselinic fatty acid remained the major fatty acid of the eight species regardless of the extraction method, the solvent used, and the origin of the samples, while the rank changed according to the maturity stage [35,36,50,51]. Nguyen, Talou, Evon, Cerny, and Merah [50] investigated the accumulation of oil and fatty acids during coriander seed maturity; the field experiment was conducted under organic cultivation conditions in Toulouse, France, for two seasons (2010 and 2011) [50]. Petroselinic and other monounsaturated fatty acids occurred in minor amounts in newly formed coriander seeds. The highest concentrations of PeFA were 74%, 34 and 59 days after flowering (DAF) during 2010 and 2011 [50]. Similar results were also reported during the 2009 season, where the PeFA amount increased from 2.8% (2 DAF) to 48.8% (12 DAF), reaching the highest level (76.4%) after 25 DAF [51]. Different results were found by Msaada, Hosni, Taarit, Hammami, and Marzouk [36] during the maturity stage of a Tunisian coriander genotype cultivated in two regions (Menzel Temime and Oued Beja, Tunisia). The results showed a higher PeFA content in the unripe seed, which increased from 40.2% (5 DAF) to 81.2% (16 DAF) in Oued Beja and from 56.3% (5 DAF) to 81.4% (33 DAF) in Menzel Temime [36]. On the other hand, aniseeds during the maturity stage showed the same trend, with lower content of PeFA in immature aniseeds and significantly higher content in mature aniseeds [35]. The maturity of coriander seeds showed opposite results for palmitic and petroselinic acid; the palmitic acid decreased, while petroselinic acid increased [36,50,51]. Msaada, Hosni, Taarit, Hammami, and Marzouk [36] reported a strong negative correlation coefficient (r = −0.98, *p* < 0.01) between the concentration of palmitic and petroselinic fatty acids. The authors linked these results with the findings of Cahoon et al., who reported that the Δ^4^-desaturation of palmitoyl-ACP to Δ^4^-hexadecenoyl-ACP with subsequent two-carbon elongation to petroselinoyl-ACP could be a possible biosynthetic pathway [66].

As might be expected, seed maturation is associated with a rise in PeFA concentration [36,50,51], while water deficit (drought effect) has been noted to cause a corresponding decrease [33,34]. For example, Laribi, Bettaieb, Kouki, Sahli, Mougou, and Marzouk [34], studying caraway, reported a decline of 12.1% and 18.4% (in comparison with the control) under moderate and strong water deficits, respectively. The same trend was reported for PeFA content in cumin seed oil under water deficit [33].

Note that the levels of PeFA can vary significantly according to the plant variety within the same species. Coşge et al. [67] reported that PeFA content in sweet fennel (*Foeniculum vulgare Miller var. dulce*) was higher (75.1%) compared to the bitter fennel (*Foeniculum vulgare Miller var. vulgare*), which contained 72.0% PeFA. Additionally, the influence of genetic variability on PeFA content in crude oil was reported in 46 genotypes of fennel by Yaldiz and Camlica [68]. Similarly, Turkish coriander varieties showed significant differences in terms of studied parameters, with PeFA rates varying between 79.7% and 81.9%, with the Erbaa variety having the highest PeFA content, while the Arslan variety recorded the lowest [48]. These studies suggest that there is potential for selecting and breeding plant varieties with high levels of PeFA.

Regarding the effect of extraction methods, Sriti, Talou, Faye, Vilarem, Marzouk, and Products [52] investigated the effect of screw configuration, nozzle diameter, and nozzle/screw distance to optimize a single-screw extruder for coriander oil extraction. The authors reported only a minor impact on the PeFA amounts extracted with the parameters mentioned (74.9% to 77.4%) [52]. Bourgou, Bettaieb Rebey, Dakhlaoui, Msaada, Saidani Tounsi, Ksouri, Fauconnier, and Hamrouni-Sellami [58] investigated the effect of green extraction methods (using MeTHF instead of hexane and supercritical CO_2_ extraction) on PeFA extraction. The authors reported that PeFA remained the major fatty acid with similar levels, 43%, 40%, and 43% using MeTHF, Sc-CO_2_, and hexane, respectively [58]. Similar results were also reported by Bettaieb Rebey, Bourgou, Detry, Wannes, Kenny, Ksouri, Sellami, and Fauconnier [44] for petroselinic levels in aniseed oil extracted with MeTHF, Sc-CO_2_, and hexane (47.0%, 48.9%, and 46.7%, respectively). The same trend was also reported for parsley when comparing the effectiveness of Sc-CO_2_ to ultrasound-assisted extraction (69.8% and 71.6% for Sc-CO_2_ and ultrasound, respectively) [65]. On the other hand, Sc-CO_2_ proved to have a higher yield of PeFA than Soxhlet/hexane for fennel seed (60.8% and 54.2%, respectively) [44]. The operating conditions of the Sc-CO_2_ (temperature, pressure, flow of CO_2_, and density of CO_2_) had no major effect on the extractability of PeFA from coriander seeds, which may offer more flexibility in adjusting different Sc-CO_2_ parameters for less energy consumption purposes [69]. Several researchers concluded that the use of green extraction methods could be considered as an alternative approach for extracting high oil content with high levels of PeFA. The only weakness of these techniques is their high prices compared to the conventional techniques [44,58,65,69].

The effect of seed roasting on the PeFA was also evaluated by Hayat, Abbas, Hussain, Shahzad, and Tahir [63]. The results revealed that microwave power (300 W, 500 W, 700 W), oven temperature (100 °C, 125 °C, 150 °C), and treatment time had a minor effect on the petroselinic content in fennel seed oil; heating with microwave at 500 W for 5 min lead to 71.3% of petroselinic (vs. 69.2% unheated) and heating with an oven at 125 °C for 20 min gave 71.2% (vs. 69.2% unheated) [63].

Fatty acids represent the largest part of the lipid fraction, so, any attempt to optimize PeFA yield should begin with optimizing the total oil yield. The Apiaceae species reported in Table 1 contained considerable quantities of lipids varying with the origin of raw material, extraction method, solvent used, and water deficit effect. The maximum lipid content of the seeds was: 30.7% (coriander), 29.1% (cumin), 29.0% (celery), 27.6% (parsley), 25.0% (anise), 20.5% (dill), 20.1% (caraway), and 20.0% (fennel). Water deficit causes a decrease of the oil content in caraway by 35.1% under moderated water deficit (MWD) and 56.5% under a strong water deficit (SWD) [34]. Similarly, cumin oil content decreased by 25.5% under MWD and 48.3% under SWD [33]. The aniseed oil content increased during the maturity stage in samples from four different countries [35]. The highest increase was reported for the Tunisian aniseeds (6.77% to 13.8% for unripe and ripe aniseeds, respectively) [35]. This development pattern of the oil content was similar to that of coriander during the maturity stage [36,50,51].

## 3. Biosynthesis of Petroselinic Acid

**Figure 2:**
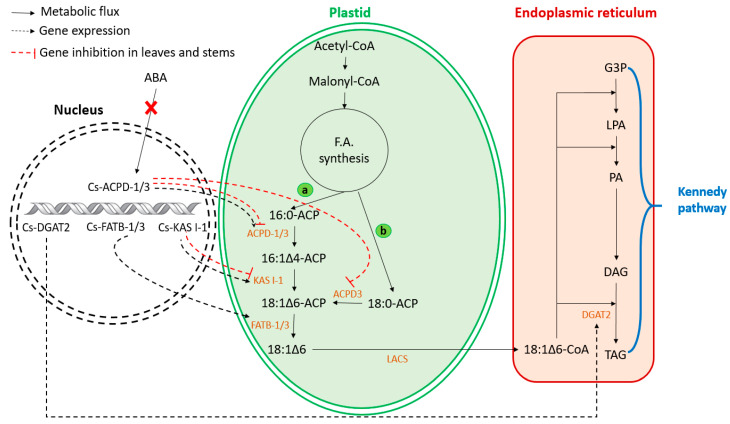
Petroselinic acid biosynthesis pathway in Umbelliferae (viz. coriander seeds). Essential enzymes are required for the biosynthesis of this acid. Initially, specific petroselinic acid biosynthesis genes (i.e., Cs-ACPD-1/3, Cs-KAS I-1) are essential for the biosynthesis of enzymes required for petroselinic acid biosynthesis. The expression of genes is not influenced by ABA. After palmitoyl-ACP (16:0-ACP) production, an ACPD1/3 is required to induce a cis double bond and produce 16:1∆4-ACP (a), while ACPD3 may desaturate 18:0-ACP (b). The elongation of 16:1∆4-ACP by KAS I-1 produced petroselinoyl-ACP (18:1∆6-ACP). The release of petroselinic acid from ACP is hydrolyzed by a fatty acyl-ACP thioesterase (FATB-1/3). Finely, petroselinic acid is activated as acyl-CoA by long-chain acyl-CoA synthetases (LACS) and transferred to the ER, and further follows the Kennedy pathway to storage as TAG.

Several species accumulate fatty acids in the form of triacylglycerol (TAG) and store them in the seeds [70]. Petroselinic acid was predominant in the TAG of Umbelliferae seeds (e.g., *Coriandrum sativum*, especially in its endosperm), whereas it was absent in the stem and the leaves [71]. Several studies confirmed the biosynthesis of PeFA via acyl-ACP pathways [72,73]. Cahoon and Ohlrogge [74] proved that petroselinic acid is not synthesized by reactions involving acyl-CoAs or by the desaturation of a fatty acid bound to a glycerolipid or CoA. The petroselinic acid biosynthesis pathway in plants is schematized in Figure 2. Cahoon, Dörmann, and Ohlrogge [72] confirmed, using an in vitro fatty acid biosynthetic system, the production of petroselinic acid with substrates generated from malonyl-CoA. In the same study, an enzyme was isolated as a 36 kDa desaturase and identified as a ∆4-acyl-ACP desaturase responsible for petroselinic acid biosynthesis. They proved that the double bond placement from the carboxyl end of acyl-ACPs was positioned by the 36 kDa desaturase. Furthermore, Yang, Li, Jia, Zhao, Taylor, Li, and Zhang [73] confirmed an exact similarity of the amino acid sequence of ACPD1 with the 36 kDa desaturase reported in previous studies [66,72,73]. Therefore, the desaturation of 16:0-ACP and 18:0-ACP by ACPD1/3 produced 16:1∆4-ACP and 18:1∆6-ACP, respectively. Thus, the elongation product of 16:1∆4-ACP allows the formation of petroselinic acid. Mekhedov, Cahoon, and Ohlrogge [71] investigated an in vitro assay with 16:1∆4-ACP as substrates to identify the enzymatic nature of the elongation reaction. They confirmed that the elongation of 16:1∆4-ACP was catalyzed by the enzyme 3-ketoacyl-ACP synthase (KAS) I-type. Thus, the elongation of 16:1∆4-ACP to 18:1∆6-ACP is regulated by KAS I-1. There are many biochemical evidences which proved the presence of petroselinic-ACP thioesterase responsible for hydrolyzing of petroselinoyl-ACP and the release of petroselinic acid from ACP [72,73,75,76]. Using long-chain acyl-CoA synthetases (LACS), petroselinic acid is exported from the plastid to the ER as acyl-CoAs [73]. The 18:1∆6-CoA is transferred to the 3rd position of DAG by DGTA2, which allows the production of TAG. In summary, the abundance and the activity of particular enzymes, namely ACPD1/3, KAS I-1, FATB-1/3, and DGAT2, explain the high content of petroselinic acid in *Coriandrum sativum* seeds [73].

One of the advantages of plant genetic engineering is the identification of *cis*-regulatory elements, which are sequences controlling gene expression at all developmental stages [77]. It has been proved that the G-box motif, AACA motifs, and Prolamin-box are essential for the quantitative expression of genes [78,79,80]. In this context, the seed’s predominant promoters of ACP (Cs-ACP1) and ∆4-palmitoyl-acyl carrier protein desaturase (Cs-4PAD) genes in developing coriander endosperms responsible for the biosynthesis of petroselinic acid have been studied to determine the regulatory mechanism of gene expression during seed development. Interestingly, various *cis*-regulatory elements, namely G-boxes such as element (ACGT), G-box (CACGCC), GCN4-like motif, AACA motifs, and Prolamin-box in the 5′-flanking regions of the Cs-ACP1 and Cs-4PAD genes, have been identified. These *cis*-regulatory elements are involved in the regulation of gene expression during seed development [81]. Further studies need to be conducted to determine the mechanism of interaction between these *cis*-regulatory elements in the regulation of Cs-ACP1 and Cs-4PAD gene expression. However, few studies have determined the transcription factors involved in the transcriptional regulation of petroselinic acid biosynthetic genes. Therefore, the identification of transcription factors and the interaction between *cis*-regulatory elements should be taken into consideration.

In addition, abscisic acid (ABA) is an important plant hormone that controls seed development and maturation and expression of several genes [82,83]. Petroselinic acid is accumulated during the flowering and seed maturity stages, which raises many questions about the hormonal regulation of petroselinic acid genes’ expression. In contrast, the expression of Cs-ACP1 and Cs-4PAD mRNA was regulated by an ABA-independent pathway [81]. Little information is available about the influence of hormones on petroselinic acid gene regulation, and further studies need to be conducted to identify the effect of different hormonal pathways interaction (viz. gibberellin) on petroselinic acid genes expression and biosynthesis.

Recently, advanced bioinformatic tools and new sequencing platforms allowed a deep understanding of biosynthesis pathways and provided information about candidate genes involved in petroselinic acid biosynthesis. Yang, Li, Jia, Zhao, Taylor, Li, and Zhang [73] studied the fatty acid profiles in the seeds of *Coriandrum sativum* L. at six developmental stages. They showed that petroselinic acid increased from 50% at the seed initiation to 76.9% at stage 6 (26 days after flowering). Furthermore, RNA sequencing using the Illumina Hiseq4000 platform, quantitative real-time PCR (q-PCR), and bioinformatic tools were used to analyze *C. sativum* seed samples at three developmental stages in order to identify candidate genes related to PeFA biosynthesis. The results showed that the genes, namely Cs-KAS I-2, Cs-FATB-1, and Cs-FATB-3, were more strongly expressed in seeds than in stems and leaves. However, Cs-KAS I-1, Cs-ACPD1, Cs-ACPD3, and Cs-DGAT2 genes were specifically expressed in seeds (Figure 2). These genes are greatly involved in the biosynthesis of PeFA. Further studies need to be conducted to investigate the expression of genes by other factors like external signal influencing (temperature, light-regulated, nutriments, etc.) and internal signal influencing (hormones, etc.).

## 4. Identification and Quantification of Petroselinic Acid

Fatty acids are commonly analyzed after conversion to their volatile fatty acid methyl esters derivatives (FAMEs), which are then separated by gas chromatography and quantified using a flame ionization detector (FID). Unfortunately, PeFA and oleic acid are usually found to be associated with the lipid fraction of Apiaceae family plants, and their separation by chromatography was considered for years as a difficult analytical challenge. The analytical methods used for quantifying both acids are based on a procedure first introduced by Von Rudloff [84]. The fatty acids are converted first to their FAMEs derivatives, then separated by thin-layer chromatography according to the degree of unsaturation. The monoenoid ester band is then recovered and analyzed by gas chromatography. Another study showed that petroselinic and oleic acids can be separated from their methyl or isopropyl esters by gas–liquid chromatography using a silica capillary column coated with 100% cyanopropyl polysiloxane stationary phase (CP Sil 88) [85]. The authors also reported that petroselinic and oleic isopropyl esters derivatives showed a better resolution than methyl esters [85]. Santinelli and Damiani [86] reported a procedure to determine the petroselinic/oleic acid ratio in oils. After trans-esterification of the fatty acids to methyl esters, the double bonds of FAMEs are epoxidized using 3-chloroperoxybenzoic acid as oxidizing agent. Subsequently, the resulting epoxy is treated with hydrochloric acid–saturated ether solution, and the oxirane ring is opened to obtain the corresponding chlorohydrin derivatives. Then the hydroxy groups are converted to their volatile silanized derivatives in the presence of hexamethyldisilazane, trimethylchlorosilane, and pyridine, and the mixture is analyzed by high-resolution gas chromatography–mass spectrometry. According to Liu and Hammond [87], phenylethyl ester derivatives showed a better resolution of oleate and petroselinate compared to methyl esters derivatives by both gas and high-performance liquid chromatography. The authors also reported that the ultraviolet absorption of the phenylethyl esters facilitates the detection of the derivatives by high-performance liquid chromatography. The phenylethyl ester derivatives were prepared by transesterification from the corresponding fatty acid using phenylethyl alcohol in the presence of sulfuric acid. A quantitative study of oleic and petroselinic acids mixture was successfully achieved by Thies [88]. Both fatty acids are converted to their butyl esters derivatives using sodium tert-butanol as a catalyst, then analyzed by gas chromatography equipped with a flame ionization detector. In another study, Avato, Fanizzi, and Rosito [16] used the same method described by Thies [88] to separate the fatty acids of some Thapsia species. In order to resolve the overlap occurring of petroselinic and oleic methyl esters of some Apiaceae family species, Knothe and Steidley [15] reported the separation and quantification of petroselinic and oleic acids from their 2-methoxyethyl ester derivatives. Additionally, the oils were analyzed as picolinyl esters to verify double bond positions. The separation and determination of the fatty acid composition of aniseed oil (including petroselinic, oleic, and vaccenic acids) were successfully achieved by Denev, Kuzmanova, Momchilova, and Nikolova-Damyanova [47]. After the conversion of fatty acids to p-methoxyphenacyl derivatives in the presence of α-bromo-p-methoxyacetophenone, the mixture was analyzed by HPLC using a converted Nucleosil 100-5SA (250 × 4.6 mm) to a silver ion form as the column and a UV detector (280 nm). Sansa-ard, et al. also evaluated the influence of alcohol lengths chain on the separation of petroselinate and oleate esters in coriander seed oil. The authors concluded, from free energy contribution calculations, that the major driving force for the separation was the interaction between the fatty acids double bond and the bulky alkyl group attached to the carboxyl group, and that the esters’ separation resolution increased with the molecular weight of alcohols by lowering the interaction between the polar carboxyl and the polar stationary phase (90%-biscyanopropyl-10%-cyanopropylphenyl polysiloxane) [89].

## 5. Isolation of Petroselinic Acid

The fatty acids are normally found in complex lipids as esters or amides. In order to isolate fatty acids, it is usually essential to release them first from their bonds by hydrolysis reactions [90]. There are several published techniques for petroselinic acid isolation. PeFA has been successfully isolated from the vegetable oil of *Coriandrum sativum* seeds with a high yield (80%) using the twin-screw extrusion. The triacylglycerols were hydrolyzed into glycerol and a mixture of fatty acids via an alkaline hydrolysis treatment using sodium hydroxide. Petroselinic acid was separated from the reaction mixture through crystallization in absolute ethanol [23]. Instead of crystallization in absolute ethanol, a pure petroselinic acid has been isolated from fennel oil by acid soap crystallization at 4 °C in methanol, followed by two urea segregations at room temperature and crystallization at −30 °C in acetone. After the acid soap crystallization, the authors noticed that the resulting crystals contained an important level of linoleic acid and palmitic acid. In contrast, the urea segregation treatment removed almost all the palmitic acid but also a part of petroselinic acid [91]. In order to isolate PeFA, Fore et al. [92] used a low-temperature crystallization of the mixed fatty acids of parsley seed oil, followed by the production of urea complexes. In addition, another approach for producing PeFA based on the selectivity of fennel oil enzymatic hydrolysis was also published by Mbayhoudel and Comeau [93]. The position of the double bond of monounsaturated fatty acids is usually established by breaking the existing ethylenic linkage with periodic acid, ozone, hydrogen peroxide, or potassium permanganate [37]. Ozonolysis of petroselinic acid produced adipic acid and lauric acid, whereas oleic acid yielded azelaic acid and pelargonic acid [84,92,94].

## 6. Reactivity of Petroselinic Acid

**Figure 3:**
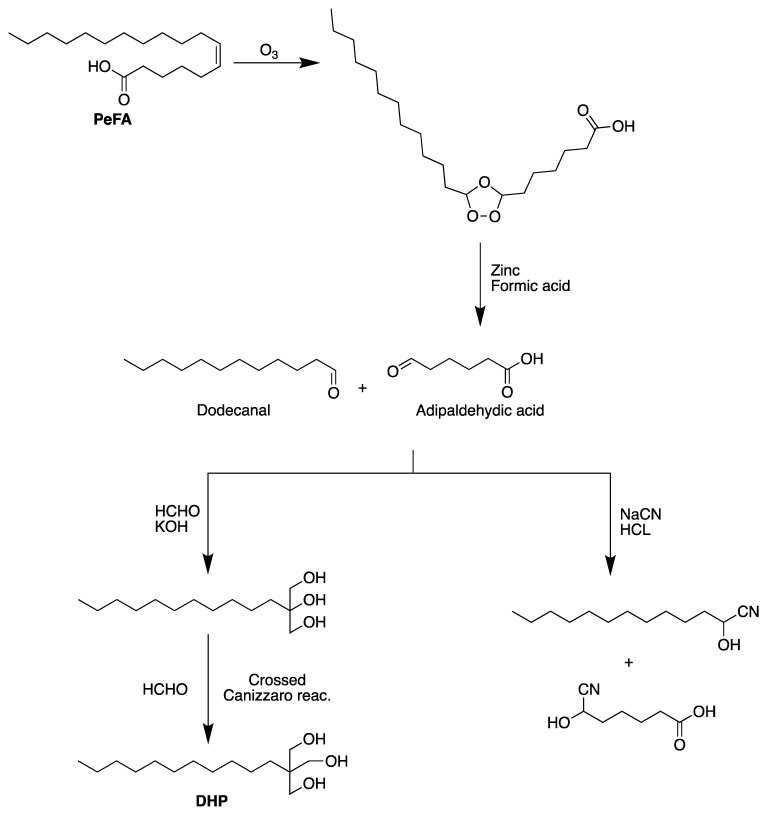
Synthesis of 2-decyl-2-hydroxymcthyl-l,3-propanediol and cyanohydrin of dodecanal from petroselinic fatty acid.

Many studies investigated the petroselinic acid reactivity in order to synthesize potentially useful derivatives of this infrequent fatty acid. In order to synthesize the 2-decyl-2-hydroxymethyl-l,3-propanediol (DHP), which could be used in the preparation of alkyd resins and plasticizers, Holmes and Moreau [95] ozonized the PeFA in the methylene chloride. The solution was emulsified with water and reacted with formaldehyde and potassium hydroxide, which yielded dodecanal and adipaldehyde acid. Afterward, dodecanal was reacted with formaldehyde and potassium hydroxide in ethanol and with formaldehyde and calcium oxide in aqueous methanol to give about 30% of DHP (Figure 3). In another study, Holmes et al. [96] used hydrogen cyanide formed in situ, and dodecanal that resulted from reductive ozonization of PeFA, to synthesize the cyanohydrin of dodecanal (in 90% crude yield) (Figure 3), which could be a multipurpose intermediate for the synthesis of various useful molecules.

**Figure 4:**
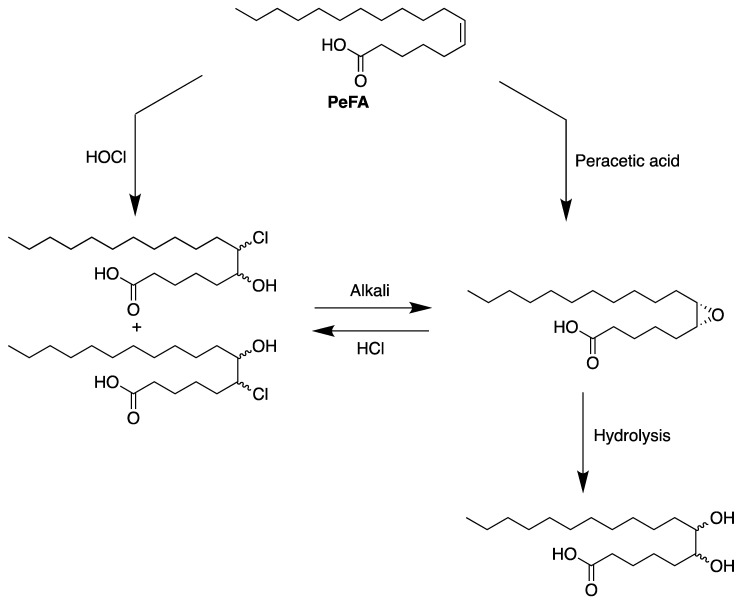
Synthesis of chlorohydroxystearic acids from petroselinic fatty acid.

In order to study the reactivity of the double bond of PeFA, Holmes et al. [97] prepared amidostearic acids from PeFA, using a variety of nitriles (hydrogen cyanide, acetonitrile, propionitrile, acrylonitrile, and benzonitrile) in the presence of sulfuric acid. Farooq et al. [98] reported the synthesis of chlorohydroxystearic acids by the action of hypochlorous acid on PeFA, and hydrochlorination of their corresponding epoxide (Figure 4). The bromohydrins and iodohydrins were also prepared.

**Figure 5:**
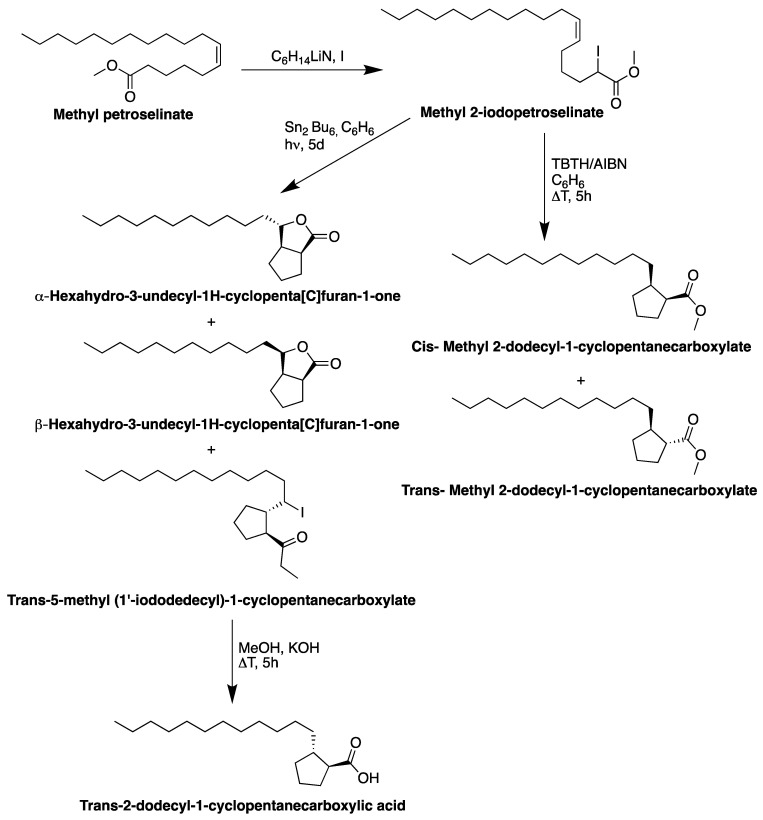
Synthesis of methyl 2-dodecyl-1-cycopentanecarboxylate from petroselinic fatty acid.

Metzger and Mahler [99] synthesized the methyl 2-dodecyl-1-cycopentanecarboxylate by cyclizing PeFA using tributylin hydride and methyl 2-iodopetroselinate (Figure 5). Afterward, the same products (methyl 2-dodecyl-1-cycopentanecarboxylate) were obtained using SnCl_2_/AgOAc as a catalyzer system. Placek and Dollear [100] synthesized some nitrogen-containing derivatives of PeFA. Petroselinonitrile was prepared by distilling the ammonolysis products of pure PeFA over phosphorus pentoxide and from parsley seed oil without first isolating the petroselinic acid. The pure petroselinonitrile was then segregated from the reaction mixture by urea complexing. Then, the petroselinonitrile was converted into primary petroselinylanmine by reduction with metallic sodium and alcohol in toluene. They have also synthesized the petroselinamide from petroselinic acid by the acidolysis of the urea complex.

A series of petroselinic acid-based estolide 2-ethylhexyl esters were prepared using a capping material varied in length and in degrees of unsaturation in the presence of perchloric acid as a catalyzer in a one-pot process, followed by an esterification process in situ. The resulting estolide 2-ethylhexyl esters showed comparable properties to the other synthesized oleic acid-based estolide 2-ethylhexyl esters with good biodegradability, which could be used as a bio-based lubricant [101]. Most surfactants are based on fatty acids with C_12_ and C_14_ chains. Due to their low solubility in water and unsuitable structure for micelle formation, fatty acids with C_18_ are barely used. In order to increase the water solubility of petroselinic acid, Dierker et al. [102] prepared a series of PeFA derivatives containing internal diols and terminal alcohols. The internal diols were synthesized by epoxidation and acid-catalyzed ring opening or by bishydroxylation with catalytic amounts of osmium tetroxide, whereas the terminal alcohols were prepared by reduction of the methyl esters. Afterward, non-ionic surfactants were prepared considering the reactivity of obtained alcohol with the p-toluenesulfonates of di-, tri-, and tetra- ethyleneglycols or by the opening of the epoxide ring using the corresponding oligoethyleneglycols. In contrast, ionic surfactants (sodium sulfate surfactants) were prepared by reacting the mono-, di- and triols with different equivalents of chlorosulfonic acid. Resulted surfactant’s properties were reported to be similar to or better than those of commercial ionic and non-ionic surfactants, C_12_ and C_14_ fatty acids based [102]. Petroselinic acid was also used for preparing biolubricant base stocks. The petroselinic acid was first converted to its methyl, isopropyl, and 2-ethyl hexyl esters derivatives; then it was converted to epoxides, followed by in situ hydroxylation and acylation using hexanoic and butyric anhydrides. The obtained acylated products showed significant lubricant properties. The products also exhibited good copper corrosion resistance. These products can be further explored for the preparation of hydraulic, metalworking, and other industrial fluid formulations [103].

## 7. Biological Properties of Petroselinic Acid

Several works have shown that PeFA displays several pharmacological activities and could be considered one of the novel pharmacological treatment strategies for several diseases. The antidiabetic activity was evaluated in vitro by Tong, Zhang, Chen, Hao, Ye, Tian, and Wu [40]. The authors showed that PeFA exhibited good inhibitory activity (IC_50_ = 6.99 mmol/L) against protein tyrosine phosphatase 1B (PTP1B), which is a negative regulator of insulin and plays a key role in developing insulin resistance in Type II diabetes mellitus. Moreover, Suzuki et al. [104] investigated the activity of saturated fatty acids (C6:0 to C22:0), cis-unsaturated fatty acids (C14:1 to C22:1), and isomers of oleic acid (C18:1) on the inhibition of topoisomerase I and II. The results showed that saturated fatty acids (C6:0 to C22:0) did not inhibit topoisomerase, but petroselinic acid and vaccenic acid (C18:1) with a cis-double bond showed strong inhibition of the enzyme. Petroselinic fatty acid acted directly on the enzyme molecule in a noncompetitive manner without DNA intercalation. Taguchi, et al. [105] studied the abilities of petroselinic acid to enhance skin penetration by determining both the in vitro skin permeability levels of indomethacin and propylene glycol and the plasma level of indomethacin after application to rat skin. They found that petroselinic acid enhanced the permeability of indomethacin and propylene glycol by a greater affinity for palmitic or stearic acid. Another investigation analyzed the antimicrobial activity of petroselinic acid against several species of bacteria (*Micrococcus pyogenes* and *Escherichia coli*), yeasts (*Saccharomyces cerevisiae*, *Candida stellatoidea*, and, *Torulopsis* sp.), and molds (*Neurospora* sp., *Alternaria* sp., *Mcor* sp., *Hormodendrum* sp., *Geotrichum* sp., *Penicilhtm* sp., and *Aspergillus* sp.) using the disc method. The results showed that petroselinic acid exhibited considerable antimicrobial activity against the majority of the tested stains [41]. The strong activity was recorded against tested bacteria, while a moderate effect was observed against mold stains, which may be used as an alternative antibacterial agent for the treatment of several infectious diseases. The authors also revealed that petroselinic acid did not inhibit the growth of the all-yeast stains tested. On the other hand, Lee, Kim, and Lee [42] assessed the antibiofilm activities of petroselinic acid at various concentrations (50, 100, 200, and 400 mg/mL) against a methicillin-sensitive *S. aureus* strain. The results demonstrated that this compound, at 100 mg/mL, inhibited *S. aureus* biofilm formation by more than 65%. Additionally, this study also demonstrated that petroselinic acid repressed the expressions of quorum-sensing-related and virulence regulator genes. The antimycotic activity of petroselinic acid was also studied by Novak et al. [106] against 22 pathogenic yeasts and molds using the filter paper disc method. The results indicated that this compound inhibited the growth of most of the yeasts and molds tested. Furthermore, it completely inhibited the growth of major periodontal pathobiont, *Porphyromonas gingivalis* at a concentration of 4–8 µg/mL via depleting the essential RagA/RagB transport machinery in *Porphyromonas gingivalis* cells by causing extracellular release of RagA/RagB-enriched OMVs.

## 8. Potential Application of PeFA

Petroselinic acid has been the subject of several patents and scientific studies. In cosmetics, PeFA is used by oral administration as a food supplement, alone or in combination with taurine and zinc gluconate, for preventing and/or fighting against aesthetic disorders of the body figure linked to modifications in the adipose tissue. In synergy with taurine and zinc gluconate, PeFA increases the amount of lipoxin A4, which is produced by the body as an anti-inflammatory agent to raise the threshold of appearance of the signals of inflammations (redness, pain, and heat) [107]. This combination of PeFA, taurine, and zinc gluconate was also reported as a functional food intended to promote weight loss and/or weight maintenance [108]. In another patent, PeFA, in association with zinc salt, was indicated to have an interesting application in keratin fiber care by improving hair quality, and preventing and controlling micro-inflammation of hair follicles [109]. PeFA salts and esters derivatives were described as potent agents to strengthen the cohesion of the skin tissue of the scalp and prevent and treat weakened or stressed scalps. Oral administration of PeFA salts esters derivatives led to preserving the underlying skin tissue and improving skin quality by decreasing collagenase and elastase activity. The authors also reported a decrease in stress and inflammation markers, such as Hsp 70 and TNF-α, that participate in skin reactivity and sensitivity and exacerbate the fragility of the skin and mucous membranes [110]. In association with α-hydroxy acids, PeFA was indicated as an anti-irritant/anti-sting agent. Glycolic acid and lactic acid were indicated to have the most effective α-hydroxy acids [111]. A cosmetic treatment of PeFA as a nailcare active compound was also indicated. The oral administration of PeFA, with a daily dose range from 3 to 1000 mg/d, could prevent and treat a nonuniform color or tint or a dull appearance of the nails [112]. Similarly, in association with taurine, arginine, cysteine, zinc, one of their salts, and lycopene, PeFA improves the micro-vascularization of the nails, in particular in a pre-hypertensive individual. PeFA was found to inhibit metalloproteinase 9 (MMP-9), which contributes to the loss of elasticity of vascular walls, abnormal distension of the vessels, and consequently, the development of a state of pre-hypertension [113]. Recently, a new application of PeFA was reported, in particular for preventing and treating intestinal ischemia-reperfusion injury. The obtained results showed that PeFA improves the intestinal tissue injury induced by intestinal ischemia-reperfusion and the survival rate of the mice. PeFA inhibits the expression of inflammatory factors [114]. Ahmad et al. used a combination of fennel seed oil, which contains 74.8% PeFA, and essential oil, which contains 70.7% trans-anethole, as functional ingredients in moisturizing cream formulations. The authors reported a positive impact on the overall structure and quality of the emulsions, including improved viscoelasticity, spreadability, and stability against oxidation, compared to the control cream. Additionally, the creams containing fennel seed oil were found to be fresher and more easily absorbed upon skin application [62].

## 9. Conclusions

Here, we summarized the different natural sources, biosynthesis, isolation, identification reactivity, benefits and pharmacological properties, and potential uses of petroselinic fatty acid. PeFA constitutes the major part of the lipid fraction of seeds from members of the Apiaceae family species; therefore, any attempt to optimize the yield of this rare fatty acid should start with the optimization of the total oil yield. Total oil yield could be optimized by choosing appropriate extraction methods and parameters. Indeed, the switch to more environmentally friendly extraction processes should be considered. Seed maturity is accompanied by an increase in petroselenic acid concentration, while water deficits (drought effect) result in a corresponding decrease. Therefore, adapting cultivation to optimal climatic conditions and using ripe, raw material, are crucial parameters for increasing petroselinic fatty acid content. The new technology in biochemistry, genetics, and molecular biology allowed us significant progress to study in deep the mechanism of petroselinic fatty acid biosynthesis pathways in seeds. Several biochemical investigations demonstrated that PeFA biosynthesis is due to a strong expression of specific genes in seed (Cs-KAS I-1, Cs-ACPD1, Cs-ACPD3, and Cs-DGAT2). Those genes are involved in the synthesis of various enzymes (such as KAS I-1 and ACPD1) that contribute to petroselinic fatty acid biosynthesis. Although the enzymes involved are identified, other mechanism pathways should be taken into consideration in PeFA biosynthesis, including the identification of transcription factors and the investigation of the expression of genes by other factors. In order to study their reactivity and biological activities, PeFA was isolated from Apiaceae seeds, and potentially useful derivatives were synthesized from this infrequent fatty acid. Petroselinic fatty acid exhibited several biological activities, including antidiabetic, antibacterial, and antifungal effects. However, further pharmacodynamics investigations on different biological targets should be carried out, as well as pharmacokinetic studies to validate its absorption, bioavailability, metabolism, and elimination. On the other hand, toxicological investigations are needed to validate its safety. In cosmetics, petroselinic fatty acid alone or in combination with other active compounds showed potential application as an anti-inflammatory agent for the treatment of skin, hair, and nail disorders.

## Figures and Tables

**Table 1:** Variation of petroselinic fatty acid content based on plant material, origin, and extraction method.

Plant Name	Origin	Extraction Method/Solvent	Oil Yield (% Based on Dry Matter Weight)	Petroselinic Acid (as % of Total Fatty Acids)	References
Anise (*Pimpinella anisum*)	Tunisia	Soxhlet/*n*-Hex	16.8	46.7	[44]
		Floch method/CHCl_3_:MeOH (2:1, *v*/*v*)	24.0	42.3	
		Soxhlet/MeTHF	23.6	48.9	
		Sc-CO_2_	≈16.5	47.0	
	Tunisia	Precellys Homogenizer/diethyl ether	25.0	31.3	[28]
	Egypt *	Percolation/CHCl_3_:MeOH (2:1, *v*/*v*)	15.3	66.2	[45]
	Tunisia *	Soxhlet/*n*-Hex	21.8	75.6	[46]
	Canada *	Soxhlet/*n*-Hex	15.8	73.2	
	Tunisia	Bligh and Dyer’s method/ CHCl_3_:MeOH:Hex (1:2:1, *v*/*v*/*v*)	6.7–13.8	13.5–46.6 (maturity)	[35]
	Serbia	Bligh and Dyer’s method/ CHCl_3_:MeOH:Hex (1:2:1, *v*/*v*/*v*)	7.1–11.9	11.1–41.3 (maturity)	
	Egypt	Bligh and Dyer’s method/ CHCl_3_:MeOH:Hex (1:2:1, *v*/*v*/*v*)	7.2–12.2	14.4–40.2 (maturity)	
	Turkey	Bligh and Dyer’s method/ CHCl_3_:MeOH:Hex (1:2:1, *v*/*v*/*v*)	7.8–13.4	10.4–45.1 (maturity)	
	Tunisia	Soxhlet/Hex (for yield calculation) Bligh and Dyer’s method/ CHCl_3_:MeOH:Hex (1:2:1, *v*/*v*/*v*) (for further analysis)	11.6	46.6	[26]
	Egypt *	Soxhlet/Hex (for yield calculation) Bligh and Dyer’s method/ CHCl_3_:MeOH:Hex (1:2:1, *v*/*v*/*v*) (for further analysis)	9.8	38.4	
	Bulgaria *	Soxhlet/*n*-Hex	n.i.	53.7	[47]
Coriander (*Coriandrum sativum*)	Turkey	Automatic analyzer/n.i	4.7–6.2	79.7–81.9 (plant variety)	[48]
	France	Soxhlet/*n*-Hex	22.9	72.6	[49]
	France	Soxhlet/*n*-cyclohexane	5.8–24.9	1.0–74.3 (maturity)	[50]
	France	Soxhlet/*n*-cyclohexane	4.6–25.1	2.8–76.4 (maturity)	[51]
	Tunisia	Soxhlet/Hex (for yield calculation) Bligh and Dyer’s method/ CHCl_3_:MeOH:Hex (1:2:1, *v*/*v*/*v*) (for further analysis)	2.7–25.9	40.2–81.2 (maturity)	[36]
	Tunisia	OMEGA 20 single-screw extruder	7.1–15.7	74.9–77.4	[52]
		Soxhlet/hexane	21.3	75.9	
	Tunisia	Precellys Homogenizer/diethyl ether	16.6	56.1	[28]
	India *	UASE /hexane	30.7 (at the optimum conditions)	76.2	[53]
	Poland *	Soxhlet/*n*-Hex	20.0	73.4	[54]
		Floch method/CHCl_3_:MeOH (2:1, *v*/*v*)	22.1	73.8	
	Korea *	Unspecific method (refer to article)	n.i.	62.0	[55]
	Egypt *	Percolation/ CHCl_3_:MeOH (2:1 *v*/*v*)	16.8	79.7	[45]
Caraway (*Carum carvi*)	Egypt *	Percolation/ CHCl_3_:MeOH (2:1 *v*/*v*)	14.3	57.6	[45]
	Canada *	Soxhlet/*n*-Hex	19.2	40.6	[56]
	Korea *	Unspecific method (refer to the article)	n.i.	34.09	[55]
	Poland *	Soxhlet/*n*-Hex	20.1	33.3	[54]
		Floch method/CHCl_3_:MeOH (2:1, *v*/*v*)	18.9	33.5	
	Tunisian	Bligh and Dyer’s method/ CHCl_3_:MeOH:Hex (4:3:2 *v*/*v*/*v*)	7.3	31.1	[27]
	Germany	Bligh and Dyer’s method/ CHCl_3_:MeOH:Hex (4:3:2 *v*/*v*/*v*)	5.8	30.8	
	Egypt	Bligh and Dyer’s method/ CHCl_3_:MeOH:Hex (4:3:2 *v*/*v*/*v*)	2.9	29.4	
	Tunisian	Bligh and Dyer’s method/ CHCl_3_:MeOH:Hex (4:3:2 *v*/*v*/*v*)	2.9–5.6	31.5–38.3 (ecotypes)	[57]
	Tunisia *	Soxhlet/*n*-Hex	13.0	43.4	[58]
		Soxhlet/MeTHF	16.0	40.3	
		Floch method/CHCl_3_:MeOH (2:1, *v*/*v*)	18.0	39.2	
		Sc-CO_2_	≈11.5	43.5	
	Tunisian	Bligh and Dyer’s method/ CHCl_3_:MeOH:Hex (4:3:2, *v*/*v*/*v*)	≈3.0, 4.0 and 7.0	28.5–35.0 (water deficit effect)	[34]
Cumin (*Cuminum cyminum*)	Tunisia	Soxhlet/Hex (for yield calculation) Bligh and Dyer’s method/ CHCl_3_:MeOH:Hex (1:2:1, *v*/*v*/*v*) for further analysis)	≈9.0, 13.0 and 18.0	43.4–55.9 (effect of drought)	[33]
	Tunisian *	Soxhlet/Hex (for yield calculation) Bligh and Dyer’s method/ CHCl_3_:MeOH:Hex (1:2:1, *v*/*v*/*v*) (for further analysis)	17.7	55.9	[59]
	Indian *	Soxhlet/Hex (for yield calculation) Bligh and Dyer’s method/ CHCl_3_:MeOH:Hex (1:2:1, *v*/*v*/*v*) (for further analysis)	15.0	41.4	[59]
	Morocco	Soxhlet/Hex	16.3–25.7	54.9–60.9 (locations)	[25]
	Lebanon *	Soxhlet/cyclohexane	23.1	49.2	[60]
	France *	Soxhlet/cyclohexane	29.1	51.5	
	Algeria *	Soxhlet/cyclohexane	13.4	51.6	
	Syria *	Soxhlet/cyclohexane	14.6	47.4	
	Korea *	Unspecific method (refer to the article)	n.i.	49.8	[55]
	Egypt *	Percolation/CHCl_3_:MeOH (2:1, *v*/*v*)	23.4	61.8	[45]
	Egypt *	Cold pressing	n.i.	41.3	[61]
Dill (*Anethum graveolens*)	Tunisia	Precellys Homogenizer/diethyl ether	15.9	87.2	[28]
	Korea *	Unspecific method (refer to the article)	n.i.	50.3	[55]
	Egypt *	Percolation/CHCl_3_:MeOH (2:1, *v*/*v*)	20.5	79.9	[45]
Fennel (*Foeniculum vulgare*)	Tunisia	Precellys Homogenizer/diethyl ether	5.8	77.8	[28]
	n.i	Soxhlet/cyclohexane	19.8	74.8	[62]
	Tunisia	Soxhlet/*n*-Hex	≈16.5	54.2	[44]
		Floch method/CHCl_3_:MeOH (2:1, *v*/*v*)	20.0	58.1	
		Soxhlet/MeTHF	18.7	61.2	
		Sc-CO_2_	≈14.0	60.8	
	Pakistan *	Petroleum ether/*n*-Hex	n.i.	69.2–71.3 (roasting)	[63]
	Korea *	Unspecific method (refer to the article)	n.i.	63.3	[55]
	Egypt *	Percolation/CHCl_3_:MeOH (2:1, *v*/*v*)	14.6	81.9	[45]
	Egypt *	Percolation/CHCl_3_:MeOH (2:1, *v*/*v*)	13.6	61.4	[56]
		UASE/CHCl_3_:MeOH (2:1, *v*/*v*)	17.9	76.4	
		Sc-CO_2_	13.7	43.1	
Parsley (*Petroselinum crispum*)	Italy	Supercritical carbon dioxide	0.4	50% (GC-FID) 181 mg/g of oil (HPLC-DAD)	[64]
		Soxhlet/*n*-Hex	n.i.	≈35% (GC-FID) 75.7 mg/g of oil (HPLC-DAD)	
	Egypt *	Percolation/CHCl_3_:MeOH (2:1, *v*/*v*)	9.7	62.8	[65]
		UASE/CHCl_3_:MeOH (2:1, *v*/*v*)	11.3	71.6	
		Sc-CO_2_	9.4	69.8	
	Canada *	Soxhlet/*n*-Hex	27.6	75.1	[56]
Celery (*Apium graveolens*)	Egypt *	Percolation/CHCl_3_:MeOH (2:1, *v*/*v*)	9.8	65.7	[45]
	Korea *	Unspecific method (refer to the article)	n.i.	49.4	[55]
	Tunisia	Precellys Homogenizer/diethyl ether	29.0	56.1	[28]
	Canada *	Soxhlet/*n*-Hex	25.4	64.3	[56]
	Egypt *	Percolation/CHCl_3_:MeOH (2:1, *v*/*v*)	9.8	58.7	[65]
		UASE/ CHCl_3_:MeOH (2:1, *v*/*v*)	14.4	75.6	
		Sc-CO_2_	8.7	61.4	

All parts used in the studies listed in this table are seeds; *: Samples purchased from local markets; authors not sure of exact origin; ≈: numerical value estimated from the diagram in the referenced article, accuracy not computed; n.i.: not indicated; CHCl_3_: chloroform, Hex: hexane, Sc-CO_2_: supercritical carbon dioxide extraction, UASE: ultrasound-assisted solvent extraction. For consistency, except where otherwise noted, all values are expressed as accurate to one decimal, regardless of uncertainties in original sources.

## Data Availability

All data were included in this manuscript.
